# Preservation of remnant esophagus during total pharyngolaryngectomy in a patient with previous subtotal esophagectomy: a case report

**DOI:** 10.1186/s40792-023-01624-9

**Published:** 2023-03-21

**Authors:** Hiroyuki Oshikiri, Hiroshi Okamoto, Yusuke Taniyama, Ryo Ishii, Akira Ohkoshi, Koreyuki Kurosawa, Michiaki Unno, Takashi Kamei

**Affiliations:** 1 Department of Surgery, Tohoku University Graduate School of Medicine, 1-1, Seiryo-Machi, Aoba-Baku, Sendai, 980-8574 Japan; 2 Department of Otolaryngology Head and Neck Surgery, Tohoku University Graduate School of Medicine, 1-1, Seiryo-Machi, Aoba-Baku, Sendai, 980-8574 Japan; 3 Department of Plastic and Reconstructive Surgery, Tohoku University Graduate School of Medicine, 1-1, Seiryo-Machi, Aoba-Baku, Sendai, 980-8574 Japan

**Keywords:** Esophageal squamous cell carcinoma, Hypopharyngeal carcinoma, Salvage surgery, Remnant esophagus, Definitive chemoradiotherapy, Indocyanine green fluorescence

## Abstract

**Background:**

With the improved survival rate of patients with esophageal cancer, secondary cancers, including pharyngolaryngeal cancer, have become a problem. Phanryngolaryngeal cancer surgery often requires esophagogastric anastomosis resection in patients with a previous history of subtotal esophagectomy. Owing to adhesions, especially surrounding the esophagogastric anastomosis, caused by the initial surgery, the second surgery might cause postoperative complications.

**Case presentation:**

A 65-year-old man was diagnosed with early stage esophageal squamous cell carcinoma and underwent endoscopic mucosal dissection. However, the histopathological depth of the tumor was pT1b, and additional treatment was required. After administration of the neoadjuvant chemotherapy, he underwent thoracoscopic esophagectomy and retrosternum reconstruction via a gastric tube (pT1N3M0 stage III). Eight months after the first surgery, tumor recurrences were observed at the anastomosis and left cervical lymph node. Definitive chemoradiotherapy was performed for the recurrences, and complete response was achieved. Seven months after chemoradiotherapy, he was diagnosed with hypopharyngeal squamous cell carcinoma in the right piriform fossa (cT2N2bM0 stage IVA), and salvage surgery was chosen as treatment. The surgical findings revealed strong adhesion around the remnant esophagus, which was difficult to dissect from surrounding tissue and was associated with a risk of breaking of the anastomosis. However, indocyanine green fluorescence imaging findings indicated sufficient blood flow to preserve the remnant esophagus, including the anastomosis, even after the interruption of blood flow from the proximal side of the esophagus by total pharyngolaryngectomy. Finally, approximately 4 cm of the remnant esophagus was preserved, and the free jejunum reconstruction with cervical vascular anastomosis was performed. Moreover, the patient was discharged without complications on postoperative day 38. After 10 months of the second surgery, a metastatic lymph node was observed in the right neck. Immune checkpoint inhibitors and chemotherapy were administered, and the patient is alive and under treatment 1.5 years after the second surgery.

**Conclusions:**

Blood supply to the remnant cervical esophagus was thought to be from the gastric conduit over the anastomosis and surrounding capillaries. Thus, the preservation of the remnant esophagus can be considered in total pharyngolaryngectomy even after < 2 years of esophagectomy by blood flow evaluation using indocyanine green fluorescence.

## Background

Owing to advances in multimodal treatment, the survival of patients with esophageal cancer, which is one of the most aggressive gastrointestinal malignancies and the sixth leading cause of cancer-related death in men, has improved [1]. Therefore, duplicate cancers detected during follow-up have often been a problem. In most cases, patients with esophageal squamous cell carcinoma (ESCC) often have head and neck squamous cell carcinoma (HNSCC) simultaneously or metachronously. It was reported that 14% of patients who undergo esophagectomy for ESCC may have HNSCC simultaneously [2]. Systemic chemotherapy, chemoradiotherapy (CRT), or surgical resection may be considered treatment strategy for esophageal cancer recurrence. However, there is no established treatment strategy for HNSCC, particularly pharyngolaryngeal cancer, developed after esophagectomy for ESCC. Surgery for pharyngolaryngeal cancer developed after subtotal esophagectomy for ESCC often requires resection of the esophagogastric anastomosis given the fact that blood flow to the remnant esophagus is interrupted. Anastomosis resection is a complex procedure owing to the adhesions caused by the initial surgery, and sternotomy may also be required in the second surgery [3]. Furthermore, salvage surgery after definitive CRT (dCRT) would be more difficult and associated with postoperative complications due to adhesions from the first surgery and the effects of irradiation [4]. We report a case of total pharyngolaryngectomy with preservation of the remnant cervical esophagus for hypopharyngeal cancer after multidisciplinary treatment, including surgery, for thoracic esophageal cancer.

## Case presentation

**Fig. 1:**
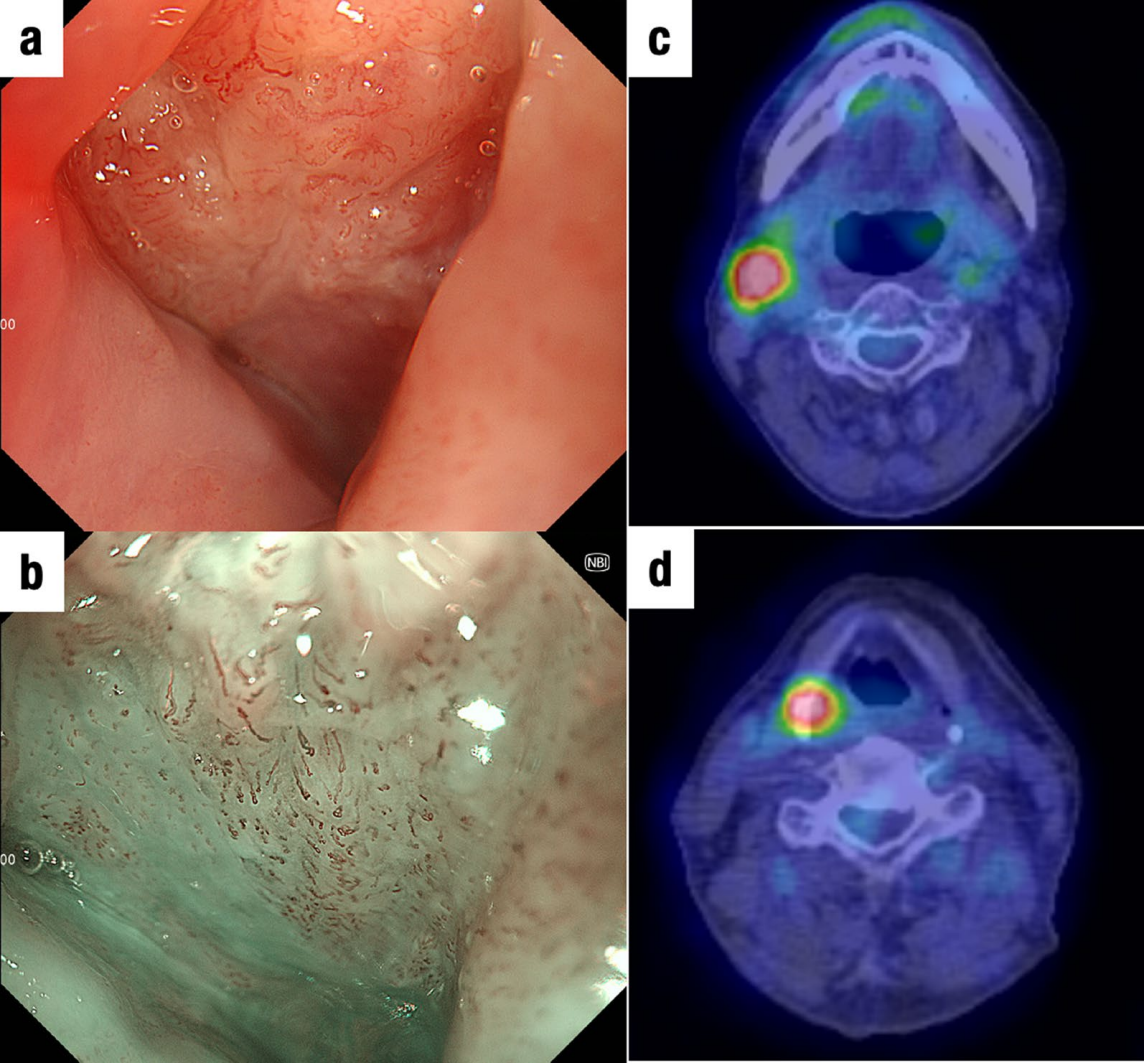
Preoperative examination. **a** Upper gastrointestinal endoscopy showing a ridge on the right piriform fossa. **b** Image-enhanced endoscopy showing an irregular granular surface and microvessel pattern in the right piriform fossa. **c** Fluorodeoxyglucose (FDG) positron emission tomography (PET) showing high FDG uptake in the right cervical lymph node. **d** FDG-PET showing high FDG uptake in the right piriform fossa

**Fig. 2:**
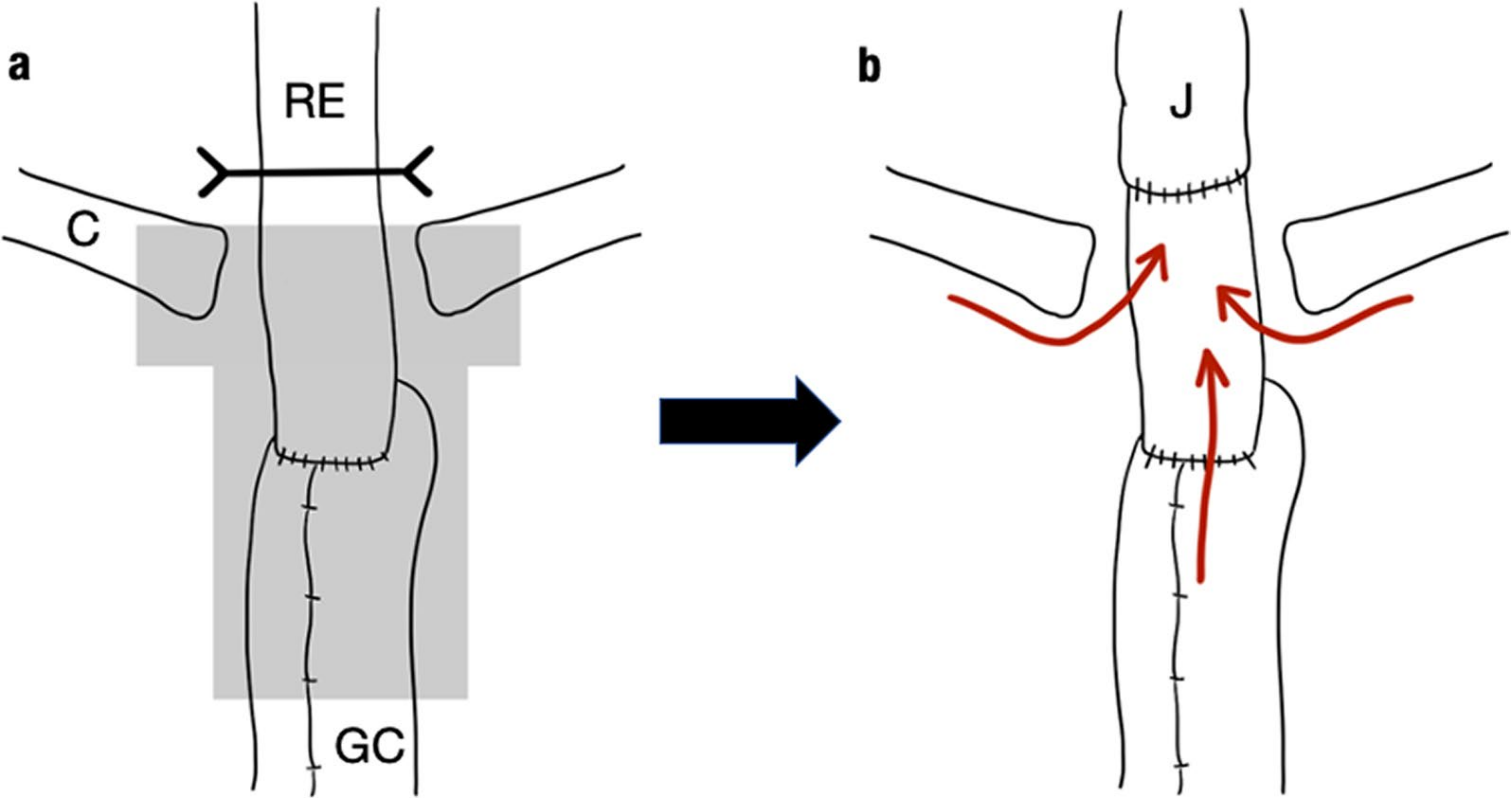
Intraoperative schema. RE: remnant esophagus, GC: gastric conduit, C: clavicle, J: jejunum. **a** There was firm adhesion around the remnant esophagus and gastric conduit on the dorsal area of the sternum (gray area). Black line is the resected line. **b** Blood flow to the remnant esophagus was thought to be nourished through the anastomosis and surrounding tissues (red arrows)

**Fig. 3:**
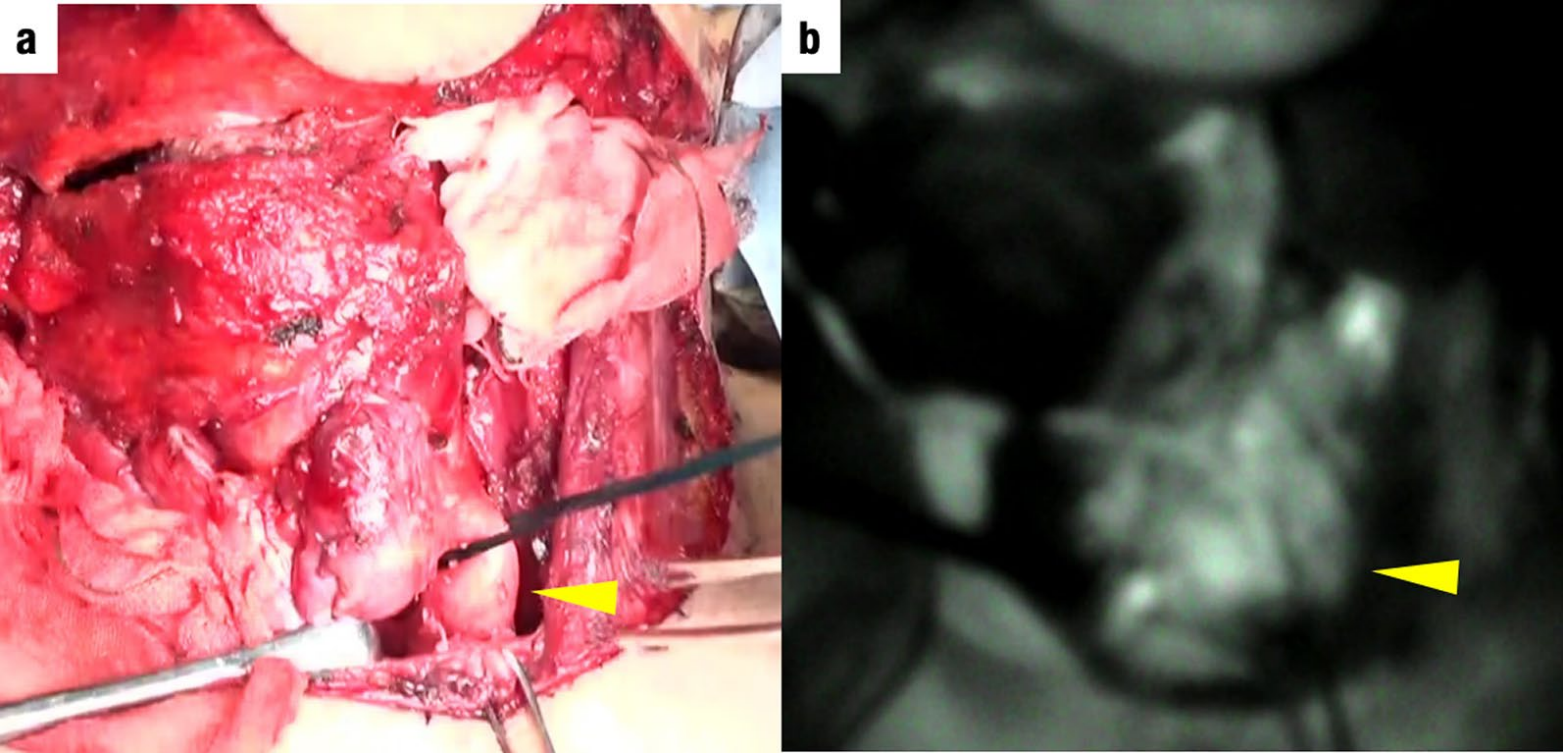
Intraoperative findings. **a** Image showing no color defects in the remnant cervical esophagus. **b** Image of the remnant cervical esophagus stained using intravenous indocyanine green after ligation of the oral side of the remnant esophagus with bilateral cervical vessels

**Fig. 4:**
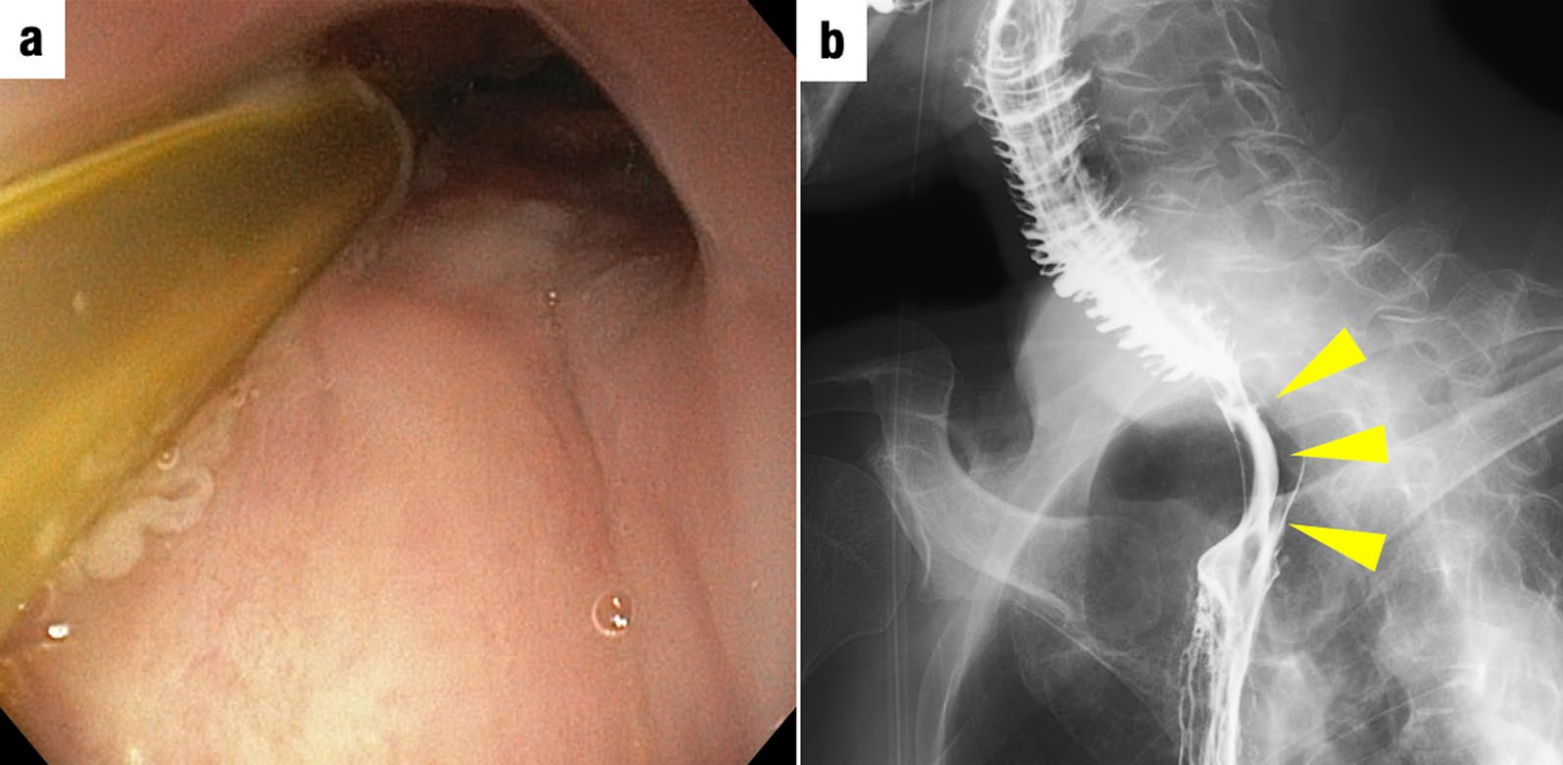
Postoperative examination. **a** Upper gastrointestinal endoscopy showing no ischemic changes in the remnant esophagus. **b** Upper gastrointestinal image showing absence of stenosis and anastomotic leakage in the remnant esophagus

We describe the case of a 65-year-old man who underwent upper gastrointestinal endoscopy for medical screening, which revealed two early stage type 0–IIc ESCC lesions 28–33 cm and 38–42 cm from the incisors. Endoscopic submucosal dissection was performed from the oral side of the lesions. The histopathological depth of the lesions was pT1b (SM2) in the eighth edition of the Union for International Cancer Control (UICC)–Tumor Node Metastasis (TNM) classification [6]. In addition, fluorodeoxyglucose (FDG)–positron emission tomography (PET)–computed tomography (CT) image revealed FDG uptake in regional lymph nodes (#2, #106, #108) and a 2-cm nodule at segment 6 of the right lung. Therefore, additional treatment was recommended according to the Guidelines for Diagnosis and Treatment of Carcinoma of Esophagus [5]. Furthermore, we decided to simultaneously resect ESCC and the lung tumor after neoadjuvant chemotherapy (NAC), because the lung tumor was suspected to be ESCC metastasis or primary lung cancer. After the administration of NAC with 5-fluorouracil (800 mg/m^2^) and cisplatin (80 mg/m^2^), thoracoscopic subtotal esophagectomy was performed; this was followed by reconstruction with a gastric conduit via the retrosternal route. The lung tumor was resected at the same time. Based on histopathological examinations under 8th UICC–TNM classification, both esophageal tumors were diagnosed as ESCCs (pT1bN3M0 stage III), and the lung tumor was diagnosed as adenocarcinoma (pT1N0M0 stage IA). After the surgery, the patient was discharged without complications. Eight months after the first surgery, local recurrence was observed at the anastomosis and the left cervical lymph node. dCRT with 5-fluorouracil (700 mg/m^2^) and cisplatin (70 mg/m^2^) combined with 60-Gy radiation for supraclavicular and superior mediastinal lesions was performed as curative treatment, and post-treatment CT scan revealed a complete response. Follow-up endoscopy performed 7 months after CRT revealed a ridge in the right piriform fossa that was diagnosed as hypopharyngeal carcinoma (Fig. 1a, b). CT and FDG-PET also showed a right cervical lymph node swelling with high FDG uptake (Fig. 1c, d). Radical resection was chosen as treatment, because the tumor and lymph node metastases were localized. We collaborated with head and neck, esophageal, and plastic surgeons to perform total pharyngolaryngectomy. After bilateral cervical lymph node dissection, the cervical esophagus was found to be controlled. To prevent injury to the anastomosis, minimal adhesiolysis of the dorsum of the esophagus was performed. However, the esophagus was firmly attached behind the sternum by adhesions (Fig. 2a). Although sternotomy was necessary to resect the anastomosis, we used indocyanine green (ICG)-based photodynamic diagnosis to determine whether the remnant cervical esophagus could be retained in place. The proximal side of the remnant esophagus was ligated with the left superior and inferior thyroid arteries; subsequently, 5 mg of ICG was injected. The remnant cervical esophagus was illuminated using a near-infrared laser beam from a thoracoscopic system (Karl Storz, Germany) (Fig. 3). We planned to preserve the remnant esophagus with the anastomosis. One hour after pharyngolaryngeal resection, an additional 5 mg of ICG was injected again, and we were able to confirm blood flow to the remnant esophagus. Therefore, we preserved approximately 4 cm of remnant esophagus and performed reconstruction with free jejunum using vascular anastomosis and permanent tracheostomy (Fig. 2b). The total operation time was 618 min, and the blood loss was 306 g. Based on the TNM criteria, the tumor was classified as stage IVA (pT2N2bM0) [6]. After 14 days of surgery, upper gastrointestinal endoscopy revealed good coloration of the mucosa (Fig. 4a) and no anastomotic leakage or stricture (Fig. 4b). The patient resumed oral intake on the same day as the endoscopy procedure, and he was discharged without complications on postoperative day 38. A metastatic lymph node was observed in the right neck 10 months after the second surgery. Immune checkpoint inhibitors and chemotherapy, including pembrolizumab (200 mg/body), 5-fluorouracil (700 mg/m^2^), and cisplatin (70 mg/m^2^), were administered, and the patient is alive and under treatment after 1.5 years of the second surgery.

## Discussion

In patients with pharyngolaryngeal or cervical esophageal cancers, preservation of voice is important for maintaining the quality of life, and intensive dCRT plays an important role in this treatment [7, 8]. However, salvage surgery is the treatment of choice for recurrence after dCRT. Because of the fragility of tissues and the strong adhesions caused by previous treatment, salvage surgery is associated with more complications than conventional surgery. Taniyama et al. reported the clinical results of 100 patients with esophageal cancer who underwent salvage surgery after dCRT with mortality and morbidity of 4.0% and 76.0%, respectively [9]. The rates of pulmonary complications and anastomotic leakage were 23.0% and 25.0%, respectively. However, after complete response was achieved, salvage surgery led to a 5-year survival rate of 46.9%, which was better than that of 13.1% in the patients with residual cancer after dCRT. In our case, although the patient had tumor recurrences in the right piriform fossa and right cervical lymph node, radical surgery was chosen as the treatment method, because the tumor was localized in the neck.

A sternotomy is often required in the second operation after esophageal cancer surgery reconstructed via the retrosternal route [3]. On the other hand, there are some reports of radical resection of head and neck cancer or esophageal cancer without sternotomy in patients after subtotal esophagectomy for esophageal cancer. Ida et al. reported seven cases, where the remnant esophagus was preserved, and tumor resection was performed in patients with hypopharyngeal or cervical esophageal cancer after subtotal esophagectomy for esophageal cancer [10]. Suga et al. also reviewed 12 patients who underwent free jejunal transfer and had a history of esophagectomy and gastric pull-up [11]. In 6 of the 12 patients, the remnant cervical esophagus was preserved. Regarding the time till the second surgery, all cases underwent second surgery after > 2 years [11]. In the present case, the second surgery was performed at 1 year and 5 months after the first surgery, and the remnant esophagus was preserved at its natural length of 4 cm. However, determining whether the remnant esophagus can be preserved is based on the patient’s condition, and an appropriate decision should be made based on ICG and other blood flow evaluations.

In recent years, ICG fluorescence imaging with a near-infrared camera has been used to evaluate blood flow in reconstructed intestinal tracts to reduce anastomotic leakage. Several reports suggest that ICG evaluation decreases anastomotic leakage rates after esophagectomy with gastric conduit reconstruction [12–14]. Although there were no cases in which imaging evaluation of blood flow to the remnant cervical esophagus was performed, four cases with gastric conduit cancer after esophagectomy could avoid total gastrectomy using the ICG fluorescence imaging [15–17]. In these cases, the tumor site was the gastric body or pylorus, and vessels around the gastric tube were ligated. ICG fluorescence imaging showed blood flow 5–7 cm from the gastric conduit esophageal anastomosis. In all four cases, subtotal gastrectomy was performed to preserve the proximal part of the gastric tube, and there were no notable complications, such as remnant tract necrosis or anastomotic leakage. Saito et al. hypothesized that revascularization in the upper region of the gastric conduit can be confirmed microscopically [15]. In our patient, the left inferior thyroid and inferior laryngeal arteries, which supply the remnant cervical esophagus, were ligated intraoperatively. When ICG fluorescence imaging showed poor blood flow, it was necessary to resect the anastomosis with an additional partial sternotomy. However, 1 h after vessel ligation, ICG fluorescence imaging still showed blood flow to the remnant cervical esophagus. Based on the abovementioned report, blood flow to the remnant cervical esophagus may have been maintained not only through the right inferior thyroid and inferior laryngeal arteries but also through the anastomosis and surrounding capillaries.

## Conclusions

Although more cases are needed to confirm our findings, intraoperative ICG fluorescence imaging can be considered useful for blood flow evaluation after preservation of remnant cervical esophagus within < 2 years after esophagectomy. ICG fluorescence imaging is a convenient, non-invasive, and useful evaluation method that can be used intraoperatively. Thus, for patients with hypopharyngeal cancer after esophagectomy, minimally invasive surgery that avoids sternotomy with the use of ICG fluorescence imaging should be considered.

## Data Availability

All data supporting this article are included in this manuscript.

## Abbreviations

FDG-PET: 18F-deoxyglucose positron emission tomography
CT: Computed tomography
CRT: Chemoradiotherapy
dCRT: Definitive chemoradiotherapy
ESCC: Esophageal squamous cell carcinoma
HNSCC: Head and neck squamous cell carcinoma
ICG: Indocyanine green
NAC: Neoadjuvant chemotherapy
TNM: Tumor Node Metastasis
UICC: Union for International Cancer Control
